# Protein tyrosine phosphatase receptor-type δ acts as a negative regulator suppressing breast cancer

**DOI:** 10.18632/oncotarget.22000

**Published:** 2017-10-24

**Authors:** Xiaotang Yu, Fan Zhang, Jun Mao, Ying Lu, Jiazhi Li, Wei Ma, Shujun Fan, Chunying Zhang, Qing Li, Bo Wang, Bo Song, Lianhong Li

**Affiliations:** ^1^ Department of Pathology and Forensic Medicine, Dalian Medical University, Dalian 116044, PR China; ^2^ Department of General Surgery, The Second Affiliated Hospital of Dalian Medical University, Dalian 116044, PR China; ^3^ Department of Human Anatomy, Dalian Medical University, Dalian 116044, PR China

**Keywords:** protein tyrosine phosphatase receptor-type δ, signal transducer and activator of transcription 3, breast cancer stem cells, epithelial-mesenchymal transition, interleukin-6

## Abstract

Protein tyrosine phosphatase receptor-type δ (PTPRD) is frequently inactivated in human cancers. This study investigated the role of PTPRD in the regulation of stemness, epithelial-mesenchymal transition (EMT), and migration and invasion in breast cancer cells. *In vitro*, PTPRD silencing using siRNA enhanced the stem cell-like properties of breast cancer cells, including their mammosphere- and holoclone-forming abilities, and it promoted tumorigenicity *in vivo*. PTPRD knockdown also increased the CD44^+^/CD24^−^ breast cancer stem cell (BCSC) population and the expression of the stem cell markers ALDH1 and OCT4. It also promoted migration and invasion by breast cancer cell, EMT, and activation of signal transducer and activator of transcription 3 (STAT3). BCSCs expressed low levels of PTPRD, displayed mesenchymal phenotypes, and were more sensitive to IL-6-mediated STAT3 activation than non-BCSCs. PTPRD expression was upregulated by IL-6 in breast cancer cells, thereby establishing a negative feedback circuit by which IL-6 induced canonical STAT3 phosphorylation and transiently upregulated PTPRD, which in turn dephosphorylated STAT3 and prevented downstream signaling via the IL-6/STAT3 cascade. These data suggest that therapies aimed at restoring or enhancing PTPRD expression may be effective in controlling breast cancer progression and metastasis.

## INTRODUCTION

Breast cancer is a significant health problem worldwide, accounting for an estimated 1.7 million new cases and 521,900 cancer-related deaths globally in 2012 [1]. Disease recurrence and metastasis are the main causes of death in breast cancer patients [2]. Histologically and molecularly, it is suggested that a small fraction of cells within tumors is responsible for cancer initiation, metastasis, relapse, and resistance to cancer therapy [3–7]. These cells are called cancer stem cells (CSCs) or cancer stem cell-like cells, and share many phenotypic and genotypic properties with somatic stem cells, such as the capacity for self-renewal and multi-potent differentiation ability. CSCs can be identified by various functional assays, including tumor sphere formation, xenograft assays, or detection of specific cell-surface markers [e.g., CD44, CD24, Oct-4, and ALDH1 in the case of breast cancer stem cells (BCSCs)] [8]. The mechanisms underlying CSC self-renewal and therapeutic resistance remain to be fully elucidated.

Protein tyrosine phosphatase receptor-type δ (PTPRD) is composed of a cell adhesion molecule-like extracellular domain and two cytoplasmic protein tyrosine phosphatase domains [9]. The *PTPRD* gene is frequently inactivated in various human cancers, including lung, colorectal, and breast cancers, glioblastoma, clear cell renal cell carcinoma, and melanoma [10–16]. Inactivating mechanisms include homozygous or heterozygous deletion, microsatellite alterations, and epigenetic silencing, suggesting that *PTPRD* is a tumor-suppressor gene [14, 16–19]. Whole exome sequencing data of 510 breast cancer specimens highlighted *PTPRD* as one of the most significantly mutated genes [15]. *PTPRD* alterations occurred in 7% of all breast cancer cases [15, 20], and the mutant *PTPRD* variant was associated with high frequencies of mutation in other genes [20]. In addition, *PTPRD* has been found to be hypermethylated in breast cancer cell lines and tissue specimens [19]. Despite the high prevalence of PTPRD inactivation in breast cancer and other tumors, the role of PTPRD in tumor progression is not yet fully understood. A previous study reported that phosphorylated signal transducer and activator of transcription 3 (pSTAT3) is a substrate of PTPRD. Accordingly, cancer-specific *PTPRD* mutations abrogated the ability of the phosphatase to dephosphorylate STAT3, leading to aberrant STAT3 activation and promotion of glioma development [18]. On the other hand, studies showed that STAT3 signaling is required for the growth of CD44^+^/CD24^−^ stem cell-like breast cancer cells [21–23]. STAT3 is a latent cytoplasmic transcription factor that serves dual functions as a signal transducer and activator of transcription, and can be activated by interleukin-6 (IL-6) and epidermal growth factor receptor (EGFR) [24]. Once phosphorylated (pSTAT3), STAT3 becomes activated, dimerizes, and translocates into the cell nucleus, where it activates gene transcription that maintains the stem cell pool, promotes cell growth and angiogenesis, and inhibits apoptosis and cell differentiation [25–28]. In addition, the IL-6/STAT3 signal pathway has been associated with induction of the epithelial–mesenchymal transition (EMT) process [29, 30].

Here we provide evidence for a negative feedback loop by which IL-6 induces canonical STAT3 phosphorylation and subsequently upregulates PTPRD, which in turn dephosphorylates STAT3 to restrain further signaling through the IL-6/STAT3 cascade. Moreover, our data suggests that low constitutive PTPRD expression in BCSCs may be a key determinant of the pluripotency and mesenchymal features of this unique population of cells.

## RESULTS

### PTPRD knockdown enhances breast cancer cell stemness

**Figure 1:**
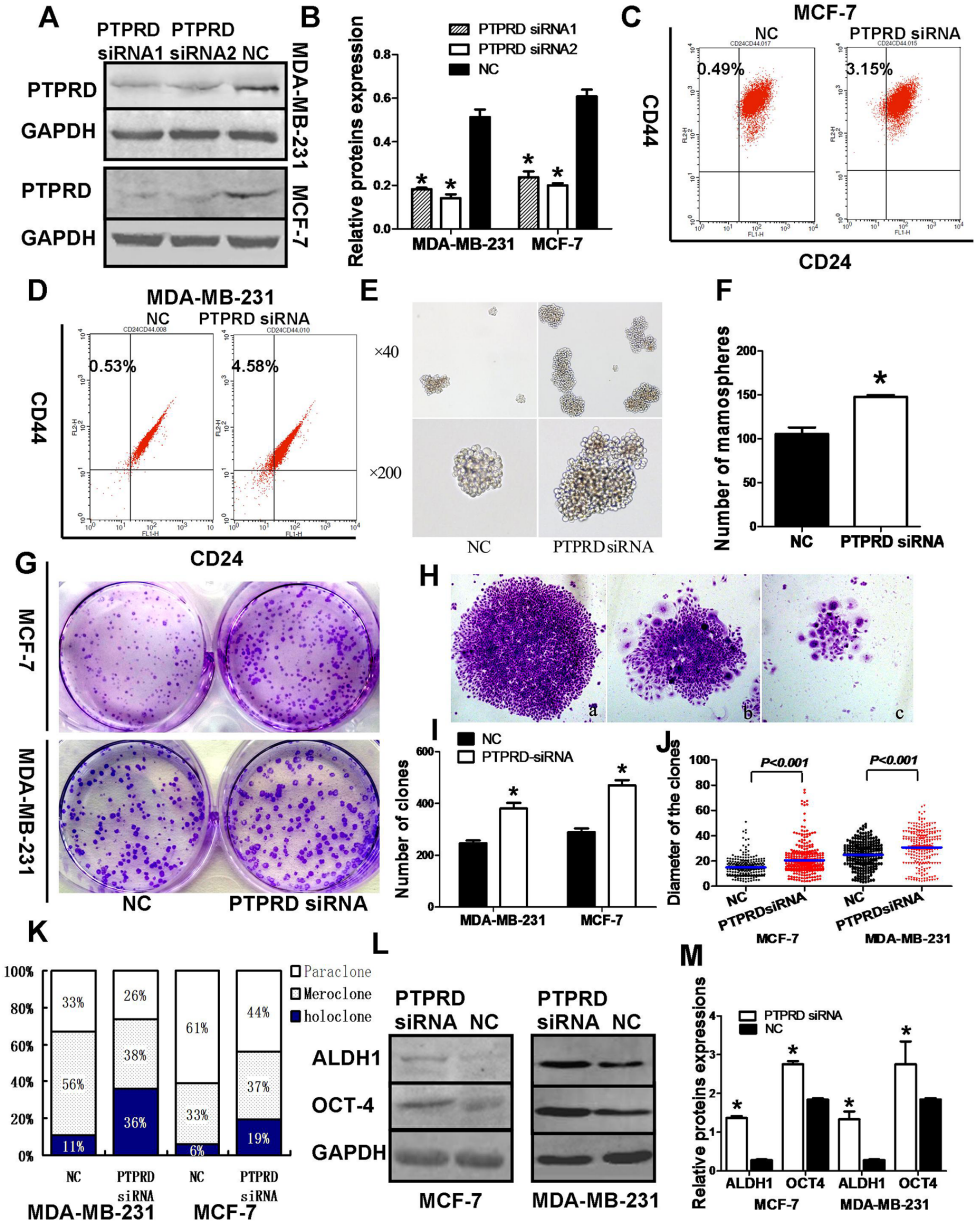
PTPRD knockdown promotes stem cell-like properties in breast cancer cells MDA-MB-231 and MCF-7 breast cancer cells were transfected with PTPRD siRNA or negative control (NC) siRNA for 48 h and then subjected to different assays. **(A)** PTPRD expression in PTPRD-silenced and NC cells. **(B)** Quantification of western blot signals from A. **(C-D)** Fluorescence cell sorting of CD44^+^/CD24^-^ cells. **(E)** Mammosphere formation assay. **(F)** Mammosphere formation quantification (^*^P < 0.05). **(G)** Holoclone colony formation assay. **(H)** Clone morphologies: a, Holoclone, b, Meroclone, c, Paraclone. **(I)** Colony formation quantification. Histograms indicate mean clone numbers formed by 500 starting cells. **(J)** Clone diameter summary data. Each dot represents an individual clone; lines indicate median diameter. **(K)** Percentual distribution of holoclones, meroclones, and paraclones formed by PTPRD siRNA- and NC siRNA-transfected cells (^*^P < 0.05). **(L)** Western blot analysis of stem cell markers ALDH1 and OCT-4. **(M)** Quantification of ALDH1 and OCT-4 levels from western blots like those shown in L (^*^P < 0.05).

To define the molecular functions of PTPRD in breast cancer, we first performed transient small interference RNA (siRNA)-mediated PTPRD knockdown in MDA-MB-231 and MCF-7 cells (Figures 1A-1B). We then assessed the effects of PTPRD downregulation on CD44^+^/CD24^−^ BCSC numbers as well as on their mammosphere- and holoclone-forming abilities. Results showed that the proportion of CD44^+^/CD24^−^ BCSCs was significantly increased after PTPRD siRNA transfection (Figures 1C-1D). As mammosphere formation is a typical BCSC property reflecting the self-renewal potential of these cells [31], we carried out mammosphere formation assays that showed that PTPRD knockdown significantly increased the number and size of spheres formed by BCSCs derived from MCF-7 and MDA-MB-231 cells (P < 0.01; Figures 1E-1F). Holoclone formation is another typical property of CSCs [32]. We cultured BCSCs in monoclonal fashion after siRNA transfection (Figure 1G) and then counted the resulting holoclones, meroclones, or paraclones based on their different morphologies (Figure 1H). Holoclones appeared as clusters of homogeneously and tightly packed small cells with regular and smooth margins (Figure 1Ha) [32]. In contrast, paraclones consisted of dispersed, larger cells with fragmented borderlines (Figure 1Hc), while meroclones exhibited an intermediate morphology (Figure 1Hb). More and larger clones were formed by breast cancer cells transfected with PTPRD siRNA than by the cells transfected with NC siRNA (P < 0.01; Figures 1I-1J). Also, the ratio of holoclones was significantly higher in the PTPRD knockdown group than in control cells (P < 0.01; Figure 1K).

At the protein level, PTPRD knockdown significantly increased the expression of the stemness markers ALDH1 and OCT-4, compared with BCSCs transfected with NC siRNA (P < 0.05; Figures 1L-1M).

### PTPRD knockdown promotes breast cancer cell migration, invasion, and epithelial–mesenchymal transition

**Figure 2:**
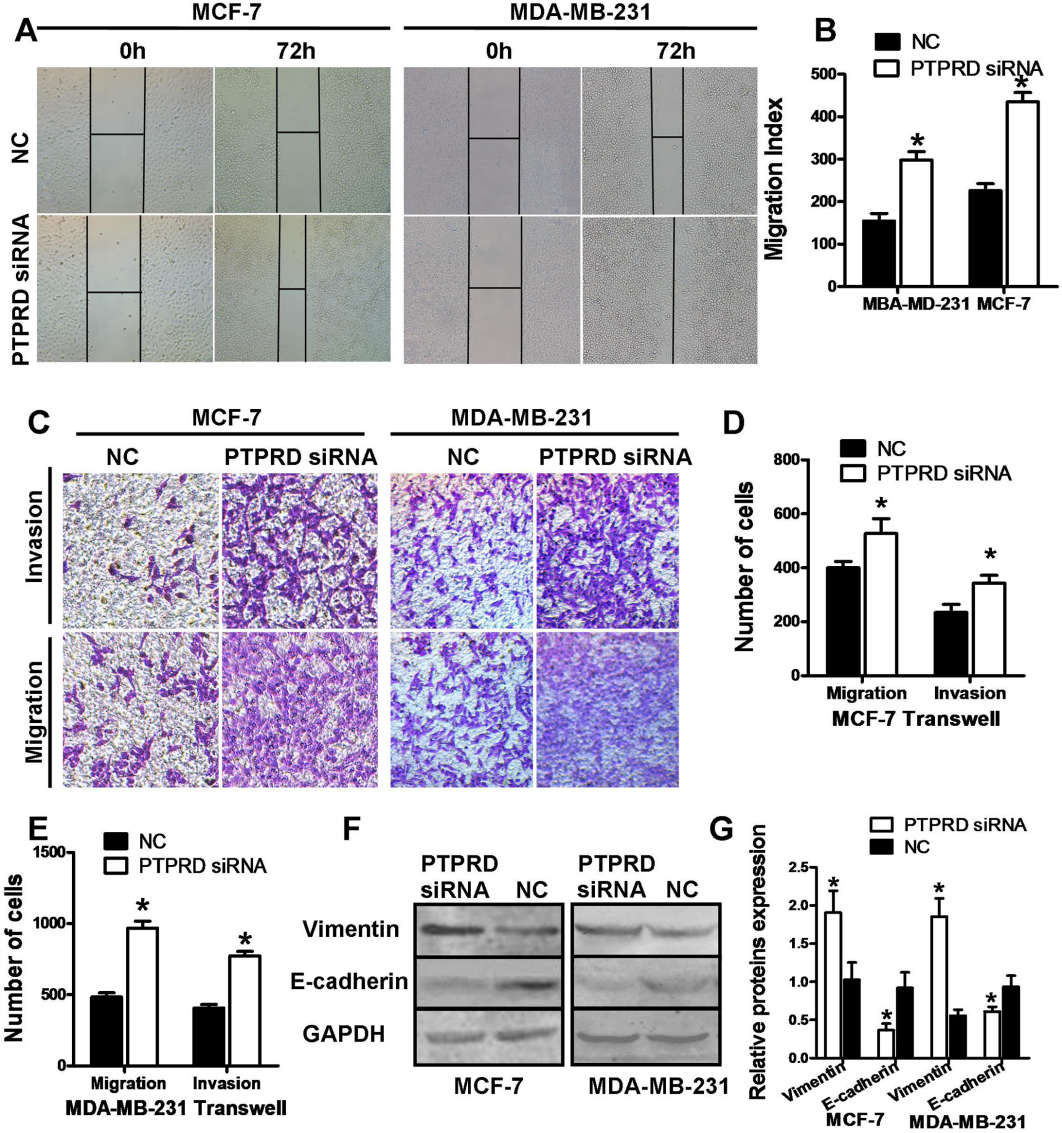
PTPRD knockdown increases migration and invasion in breast cancer cells MDA-MB-231 and MCF-7 cells were transfected with PTPRD or NC siRNAs for 24 h, and then subjected to different assays. **(A)** Representative microphotographs of the wound-healing assay. **(B)** Wound-healing assay quantification data. **(C)** Representative microphotographs of tumor cell migration and invasion assays. **(D)** Cell migration and invasion assay quantification of MCF-7. **(E)**. Cell migration and invasion assay quantification of MDA-MB-231. **(F)** Western blot analysis of vimentin and E-cadherin expression. **(G)** Protein expression quantification from western blots like those shown in F.

Proliferation and cell cycle distribution analyses in breast cancer cells showed no significant differences between PTPRD-knockdown and control cells (Supplementary Figures 1 and 2). In contrast, wound-healing assays indicated increased motility in PTPRD-knockdown cells compared with control cells (P < 0.05; Figures 2A-2B). Transwell assays, on the other hand, showed that migration and invasion capacities were also significantly increased in cells transfected with PTPRD siRNA (P < 0.01; Figures 2C-2E). These data clearly indicate that downregulation of PTPRD promotes migration and invasion in breast cancer cells.

Next, we analyzed the effect of PTPRD silencing on EMT markers using western blot. Consistent with EMT induction, the expression of the epithelial marker E-cadherin was decreased, while the expression of the mesenchymal marker vimentin was increased, in PTPRD-silenced cells (P < 0.05; Figures 2F-2G).

### PTPRD negatively regulates IL-6/STAT3 signaling

**Figure 3:**
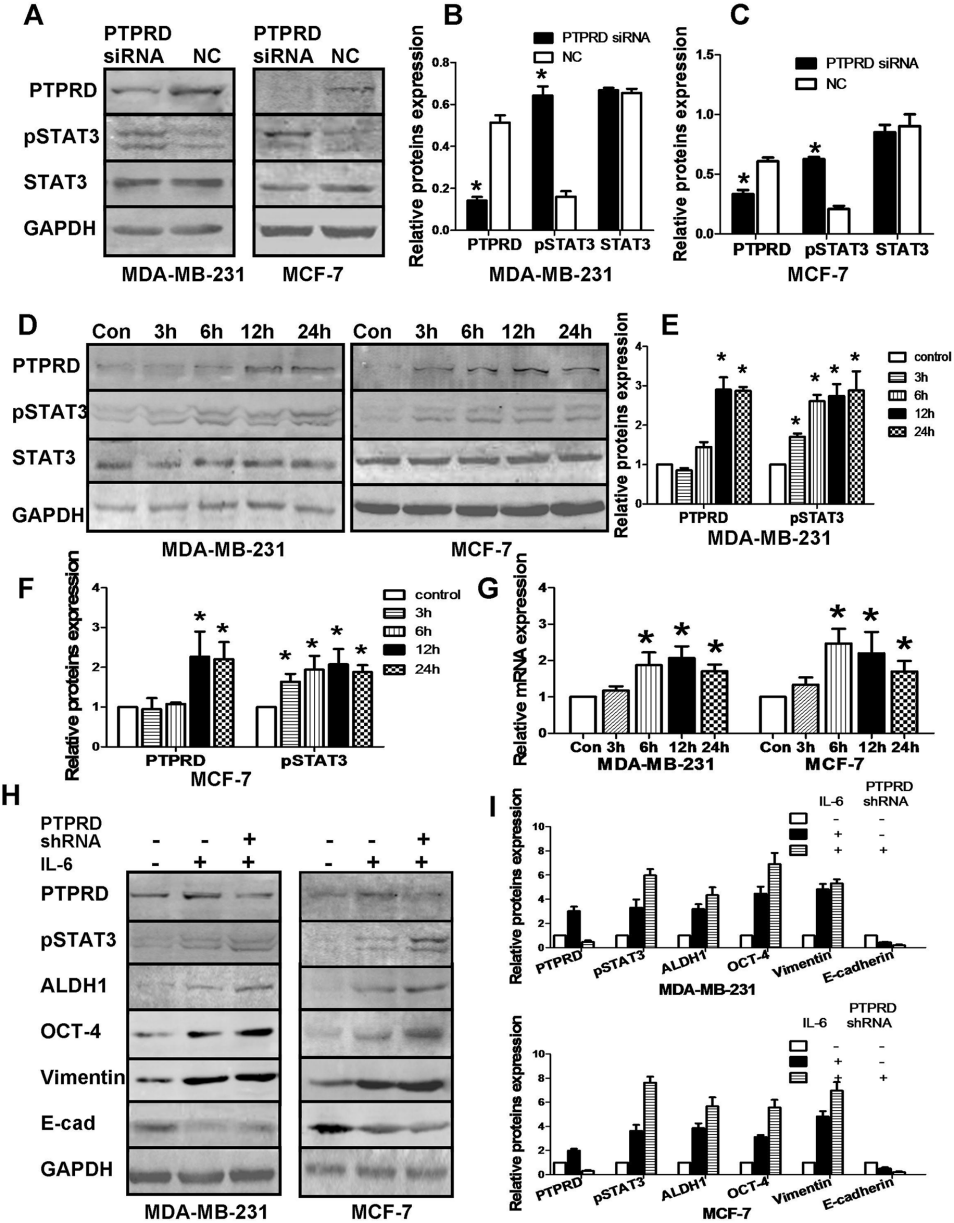
PTPRD regulates the IL-6/STAT3 signaling pathway **(A)** Western blotting analysis of PTPRD, STAT3, and pSTAT3 in breast cancer cells transfected with PTPRD or NC siRNAs for 48 h. **(B-C)** Quantification of western blot data from A (^*^P < 0.05). **(D)** Western blot analysis of PTPRD, STAT3, and pSTAT3 in breast cancer cells treated with IL-6. **(E-F)** Quantification of western blot data from D. **(G)** Time course of PTPRD mRNA induction in IL-6-treated cells, determined by qRT-PCR (^*^P < 0.05). **(H)** Western blot analysis of PTPRD, pSTAT3, and stemness and EMT markers in breast cancer cells transfected with PTPRD or NC shRNAs. IL-6 was added to cultures for 12 h (PTPRD and pSTAT3) or 48 h (vimentin, E-cadherin, ALDH1, and OCT4) before expression analyses. **(I)** Protein quantification from western blots like those shown in H (^*^P < 0.05).

The IL-6/STAT3 signaling pathway plays an important role in the regulation of stemness, EMT, and metastatic dissemination of cancer cells [21, 29, 30]. We therefore investigated whether the positive influence of PTPRD on breast cancer cell stemness and EMT depends on IL-6/STAT3 signaling. Our results showed that PTPRD knockdown significantly increased pSTAT3 levels, without affecting total STAT3 (Figures 3A-3C). To further analyze this interaction, we first activated STAT3 by treating cells with IL-6 (20 ng/ml) in serum-free conditions and analyzed samples 3, 6, 12, and 24 h later. We found that both pSTAT3 and PTPRD protein levels were significantly induced by IL-6 exposure (Figures 3D-3F). Specifically, PTPRD expression in both MDA-MB-231 and MCF-7 breast cancer cells increased significantly 12 to 24 h after IL-6 treatment. PTPRD mRNA levels were also increased in IL-6-treated cells (Figure 3G).

Concomitantly, IL-6 exposure increased the expression of the stem cell markers OCT-4 and ALDH1 in cells, and suggested EMT activation by increasing vimentin and repressing E-cadherin expression (Figures 3H-3I).

These results provide evidence that IL-6 induces relatively rapid and transient PTPRD expression, suggesting a potential role for PTPRD as a negative feedback regulator of IL-6/STAT3 signaling. To test this possibility, we evaluated IL-6-mediated STAT3 phosphorylation in both control and PTPRD-silenced breast cancer cells. Indeed, western blot results showed relatively higher pSTAT3 levels in PTPRD-knockdown cells (Figures 3H-3I). In addition, increased levels of ALDH1, OCT-4, and vimentin, and decreased E-cadherin expression, were observed in IL-6-exposed, PTPRD-knockdown cells compared with PTPRD-competent cells similarly stimulated with IL-6 (Figures 3H-3I).

These data suggest that PTPRD is an IL-6–induced negative-feedback regulator that prevents overactivation of the IL-6/STAT3 signaling pathway.

### BCSCs express low PTPRD levels and are more sensitive to IL-6 stimulation

**Figure 4:**
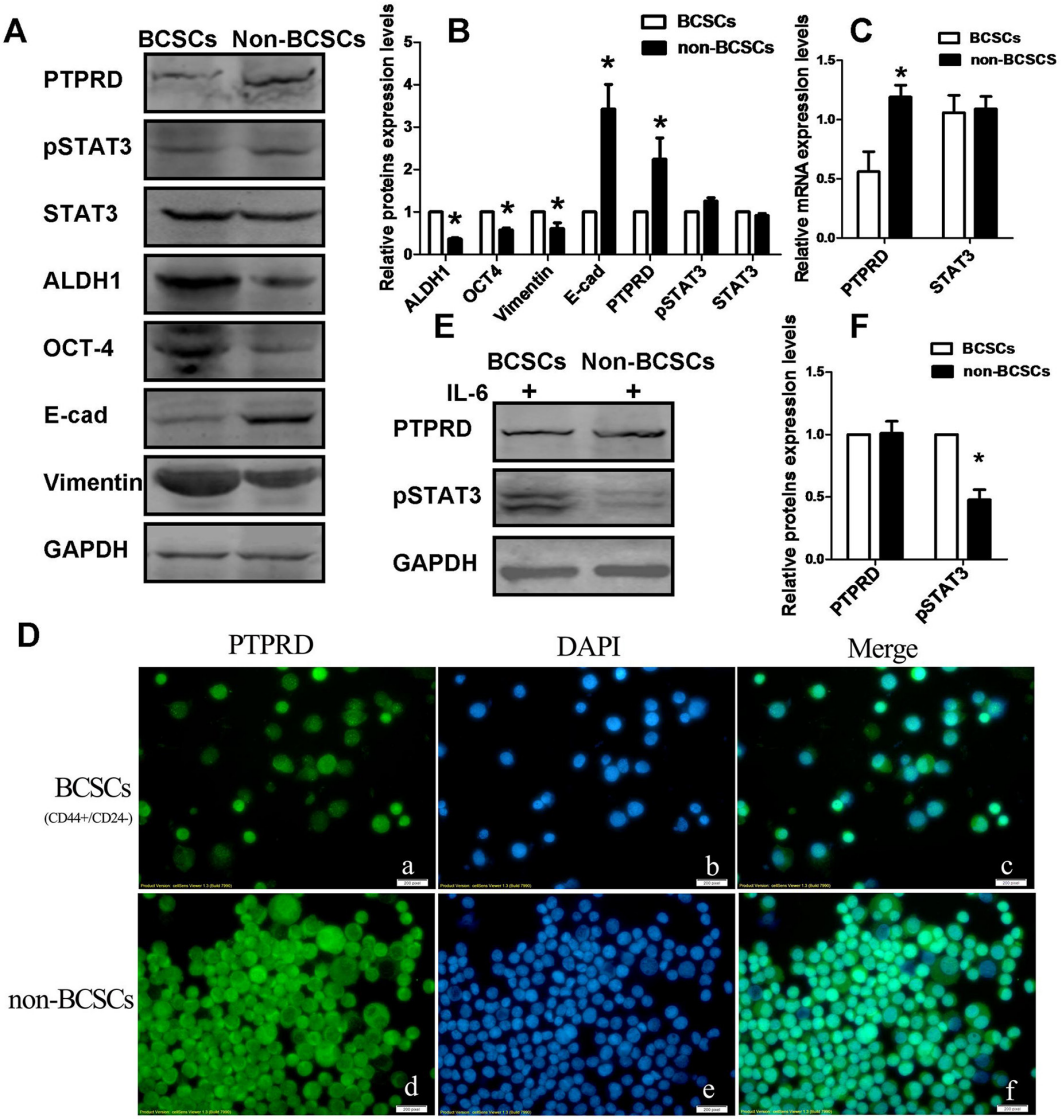
Endogenous PTPRD, STAT3, pSTAT3, E-cadherin, vimentin, ALDH1, and OCT4 levels in BCSCs and non-BCSCs **(A)** Representative western blots. **(B)** Protein quantification from western blots like those shown in A (^*^P < 0.05). **(C)** Quantification of mRNA in CD44^+^/CD24^-^ BCSCs and non-BCSCs using qRT-PCR (^*^P < 0.05). **(D)** Immunofluorescence staining of PTPRD in serum-starved CD44^+^/CD24^−^ BCSCs and in non-BCSCs. **(E)** Western blotting analysis of PTPRD and pSTAT3 in BCSCs and non-BCSCs. MDA-MB-231 cells were stimulated with IL-6 for 12 h before separation of BCSCs and non-BCSCs. **(F)** Quantification of western blot experiments like those shown in E (^*^P < 0.05).

To assess the expression of PTPRD in CD44^+^/CD24^−^ BCSCs and in non-BCSCs, BCSCs were isolated from MDA-MB-231 cells by using magnetic beads. To eliminate the effect of cytokines and growth factors that may activate STAT3 and affect the expression of PTPRD, the cells were serum-starved for 24 h before isolation. Expression of the stem cell markers ALDH1 and OCT-4 was then detected using western blotting to assure successful isolation of BCSCs. Our data confirmed that CD44^+^/CD24^−^ BCSCs expressed significantly elevated levels of ALDH1 and OCT-4 (Figures 4A-4B). Endogenous PTPRD and STAT3 mRNA expression was then estimated by qRT-PCR. Results showed that BCSCs expressed significantly lower levels of PTPRD mRNA compared with non-BCSCs, while STAT3 mRNA levels were similar in both cell types (Figure 4C). Furthermore, we profiled the expression of PTPRD, STAT3/pSTAT3, E-cadherin, and vimentin in BCSCs and non-BCSCs using western blot. Consistent with mRNA results, data showed that PTPRD expression was downregulated in CD44^+^/CD24^−^ BCSCs, whereas levels of STAT3 and pSTAT3 were comparable for both BCSCs and non-BCSCs. In addition, expression of vimentin was significantly increased, while that of E-cadherin was decreased, in BCSCs compared to non-BCSCs, suggesting that these CSCs display mesenchymal characteristics (Figures 4A-4B). Immunofluorescence staining in cultured cells further confirmed reduced PTPRD expression in BCSCs (Figure 4D).

Because STAT3 signaling is more active in PTPRD-silenced cells, and BCSCs showed constitutively lower PTPRD levels, it was of interest to determine whether STAT3 activation in response to IL-6 occurs more readily in BCSCs than in non-BCSCs. To assess this, cells were starved for 24 h, and then stimulated with IL-6 for 12 h. Then, BCSCs were isolated and the expression of pSTAT3 and PTPRD was measured by western blot in BCSCs and non-BCSCs. Results showed that pSTAT3 was significantly higher in BCSCs than in non-BCSCs (Figures 4E-4F). This result indicates that low baseline expression of PTPRD correlates with increased IL-6 sensitivity and enhanced STAT3 signaling in BCSCs.

### PTPRD silencing promotes tumor xenograft growth *in vivo*

**Figure 5:**
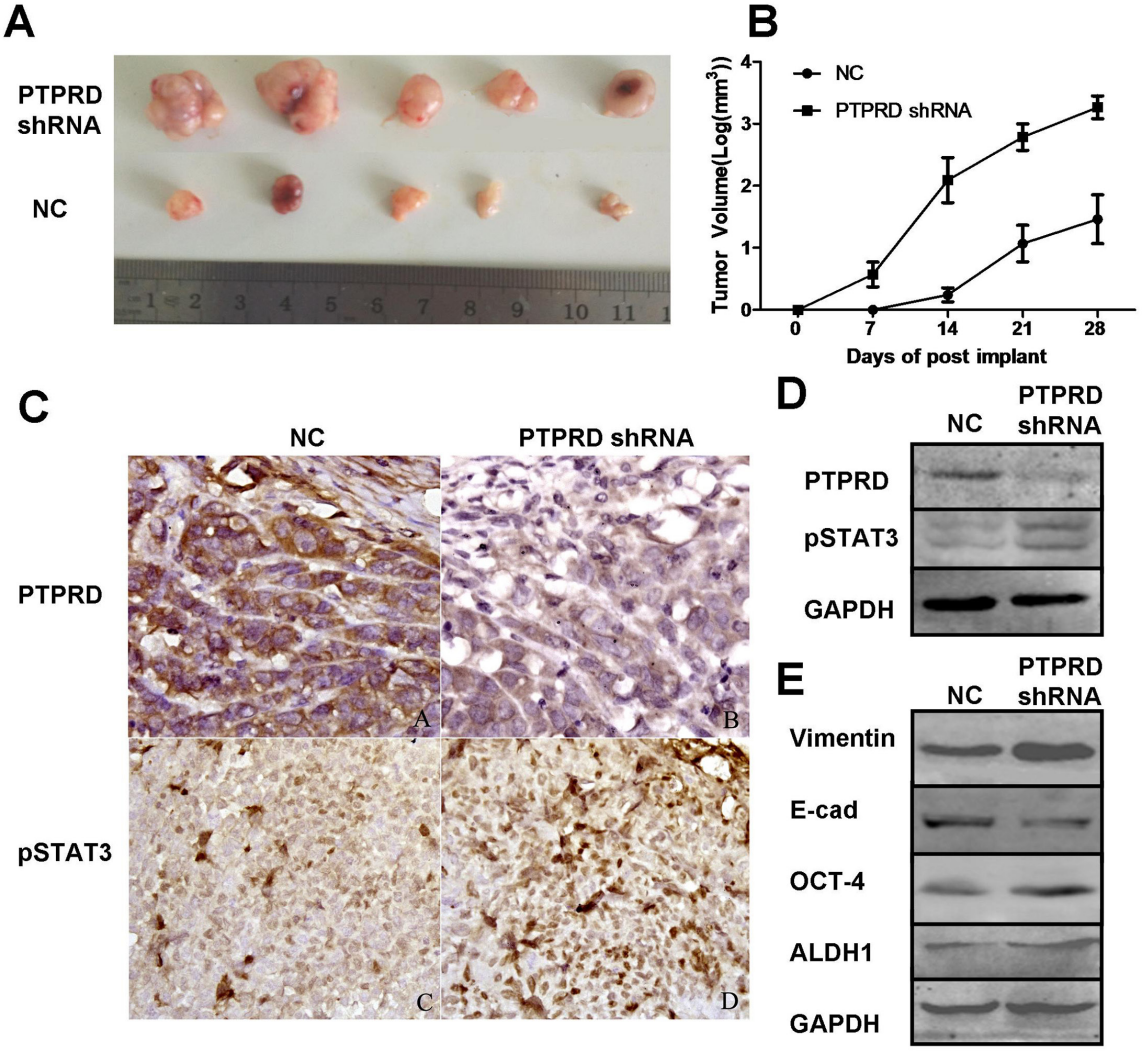
PTPRD knockdown promotes breast cancer cell xenograft formation and growth in nude mice **(A)** Pictures of tumor xenografts excised from nude mice on day 28 post-implantation. **(B)** Tumor xenograft growth curves. Tumor size was measured every other day up to 28 days (P < 0.05). Data are expressed as mean ± SEM. **(C)** Immunohistochemical detection of PTPRD and pSTAT3 in tumor xenografts. **(D)** Western blot analysis of PTPRD and pSTAT3 in tumor xenografts. **(E)** Western blot analysis of E-cadherin, vimentin, ALDH1 and OCT-4 in tumor xenografts.

To assess the tumorigenic effect of PTPRD downregulation, we generated an *in vivo* breast cancer xenograft model by subcutaneously injecting breast cancer cells MDA-MB-231 that had been transfected with PTPRD shRNA or NC shRNA into the mammary glands of SCID mice. Tumor volume measurements showed that PTPRD downregulation significantly promoted xenograft formation and growth (Figures 5A-5B). On the other hand, western blotting and immunohistochemistry analyses indicated decreased PTPRD and increased pSTAT3 expression in samples from PTPRD shRNA-transfected breast cancer cells, compared with NC shRNA controls (Figures 5C-5E).

## DISCUSSION

Although PTPRD inactivation is highly prevalent in various human cancers [33–36], its specific contribution to tumorigenesis remains poorly understood. Thus, the current study sought to investigate the signal transduction pathways influenced by PTPRD. Consistent with its previously ascribed role as a tumor-suppressor, we show that PTPRD downregulation enhances CSC marker expression and promotes migration, invasion, and EMT in breast cancer cells *in vitro*. Moreover, shRNA-mediated PTPRD silencing enhanced the growth of breast cancer cell xenografts *in vivo*. The present evidence suggests that these effects result from attenuation of IL-6/STAT3 signaling by PTPRD. Specifically, we show that in addition to mediating STAT3 activation, IL-6 induces the expression of PTPRD. This is the first study, to our knowledge, to report the effect of IL-6 on PTPRD expression. As our silencing experiments suggest, the consequence of the late increase in PTPRD after IL-6 exposure is repression of further STAT3 activation. Thus, PTPRD appears to act as a negative feedback regulator that balances IL-6/STAT3 pathway activity.

EMT is a process by which epithelial cells lose polarity and cell-cell contact, and acquire migration and invasion capacities characteristic of mesenchymal stem cells. EMT plays an important role in organ formation and cell differentiation, and occurs also during wound healing and tumor metastasis [37, 38]. Our current data showed that PTPRD downregulation significantly induced breast cancer cell EMT by promoting, in parallel with increased vimentin and decreased E-cadherin expression, their migratory and invasive capacities. The fact that PTPRD downregulation had no effect on breast cancer cell viability and cell-cycle distribution indicates that the tumor-suppressive role of PTPRD is mainly related to EMT downregulation. Our observations are consistent with two other studies. In colorectal cancer, PTPRD suppressed tumor cell migration by promoting cell-cell adhesion [17]. In glioma, loss of PTPRD expression accelerated tumor formation, but did not affect cell proliferation [18].

The molecular mechanisms underlying PTPRD regulation of BCSCs properties and EMT remain to be determined. PTPRD acts on several substrates, including desmoplakin [16] and STAT3 [18]. STAT3 is an important regulator of stem cell maintenance and function [21, 23], and its dephosphorylation by PTPRD regulates its transcriptional activity *in vitro* [12, 18, 35]. The present study showed that PTPRD knockdown significantly increased STAT3 activation *in vitro*, and promoted tumorigenesis in mice characterized also by high pSTAT3 expression in tumor cells *in vivo*. This finding suggests that PTPRD dephosphorylates STAT3 in breast cancer cells *in vivo*.

CSCs are a unique subset of cancer cells with self-renewal and a multilineage differentiation capacities [39]. Interestingly, we found that PTPRD expression is low in CD44+/CD24- BCSCs compared with non-BCSCs within the same cell lines. Moreover, our experiments revealed that siRNA-mediated suppression of PTPRD expression enhanced CSC numbers and features, i.e. increased the CD44+/CD24- subpopulation, enhanced mammosphere and holoclone forming ability, and stimulated the expression of the stem cell markers ALDH1 and OCT-4. Although a number of studies have reported or suggested a role for PTPRD in tumorigenesis [10–13, 16–18, 35], no direct evidence so far has associated PTPRD with stemness features. PTPRD belongs to the LAR subfamily of the LAR protein tyrosine phosphatase and is structurally and functionally similar to its members PTPσ and LAR [40, 41]. Quarmyne et al. demonstrated that PTPσ repressed proliferation of hematopoietic stem cells (HSCs). Compared with *PTPσ*+/+ cells, bone marrow cells from *PTPσ*−/−mice displayed a marked increase in competitive repopulating capacity [42]. Furthermore, human PTPσ- HSCs had a 15-fold higher repopulating capacity than PTPσ+ HSCs [42]. Indeed, our current data suggest that PTPRD function in BCSCs is similar to that of PTPσ on HSCs. We also detected high vimentin and low E-cadherin expression in BCSCs, consistent with findings from other studies showing that CSCs tend to have mesenchymal phenotypes [43–45].

Although PTPRD expression in BCSCs was lower than in non-BCSCs, to our surprise, pSTAT3 levels were similar in both populations. Several studies showed that STAT3 activation can promote stem cell-like properties in cancer cells [46–48]. It has been reported, also, that BCSCs exhibit higher levels of pSTAT3 [49]. The discrepancy between those and our own observations may be due to cell culture differences, as cells in this study were cultured in DMEM without FBS or cytokines for 24 h before isolation to eliminate possible effects of cytokines or growth factors on PTPRD and pSTAT3 expression. Thus, our experiments more likely reflect the basic status of STAT3 activation and PTPRD expression in quiescent BCSCs and non-BCSCs, without the influence of cytokines or growth factors. We reasoned that lower PTPRD expression in BCSCs might lead to enhanced STAT3 phosphorylation in response to IL-6, an assumption that proved to be true after measuring IL-6-induced pSTAT3 in both subpopulations. Therefore, our results suggested that in the quiescent state, STAT3 activation is similar in BCSCs and non-BCSCs, while under IL-6 stimulation, lower expression of the negative-feedback regulator PTPRD leads to stronger STAT3 activation in BCSCs.

**Figure 6:**
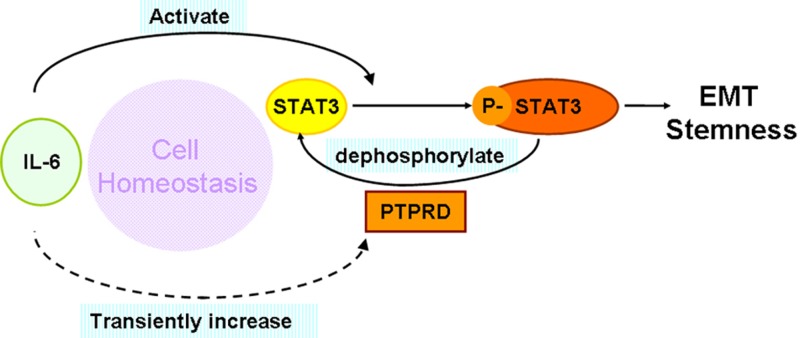
Model of the negative feedback circuit regulating the IL-6/STAT3 signaling pathway in breast cancer cells IL-6 stimulation induces STAT3 activation, promoting EMT and stemness. In parallel, IL-6–induced PTPRD expression leads to STAT3 dephosphorylation to curb the overactivation of STAT3 signaling. Loss of PTPRD function in breast cancer cells leads to overactivation of STAT3 and promotes tumorigenesis.

In conclusion, we propose that PTPRD is an IL-6–induced negative regulator of the IL-6/STAT3 signaling pathway (Figure 6). In normal cells, this feedback circuit contributes to the maintenance of cell homeostasis; in contrast, in cancer cells loss of PTPRD expression or function leads to STAT3 overactivation and promotion of tumorigenesis. Therapies aimed at restoring or enhancing PTPRD expression and/or activity may be effective in controlling breast cancer progression and metastasis.

## MATERIALS AND METHODS

### Cell lines and culture

The human breast cancer cell lines MDA-MB-231 and MCF-7 were obtained from the Cell Bank of Chinese Academy of Sciences (Shanghai, China) and cultured in DMEM/F12 medium (HyClone, Logan, UT, USA), supplemented with 10% fetal bovine serum (FBS; Gibco Laboratories, Grand Island, NY, USA) in a humidified incubator with 5% CO_2_ at 37°C.

To assess phenotypic and gene expression effects of IL-6, MDA-MB-231 and MCF-7 cells were cultured in serum-free medium containing 20 ng/ml of IL-6 (Cat #200-06, Peprotech, Rocky Hill, NJ, USA) and harvested at 3, 6, 12, 24, and 48 h. Cells cultured in serum-free medium without IL-6 were used as control.

### PTPRD siRNA transfection

PTPRD-targeted and negative control (NC) siRNAs were synthesized by Invitrogen (Carlsbad, CA, USA). The siRNA oligonucleotides targeting PTPRD were as follows: (siRNA#1, 5'-AUUGACAGCAACAACCCUGTT-3' and 5'-CAGGGUUGUUGCUGUCAAUTT-3'; siRNA#2, 5'-UUUGAAGUUUAGUGGCUGCTT-3' and 5'-GCAGCCACUAAACUUCAAATT-3'; and siRNA#3, 5'-AUUUCAUGAUUAGUGGGUGTT-3' and 5'-CACCCACUAAUCAUGAAAUTT-3'). A short hairpin RNA (shRNA) expression plasmid (pGPHI/GFP/Neo-PTPRD) was designed by GenePharma (Shanghai, China). After DNA sequence confirmation, plasmids (final concentration = 50 nM) were transfected into breast cancer cells using Lipofectamine 2000 (Invitrogen) according to the manufacturer's instructions.

### Immunofluorescence

Cells were fixed in 4% paraformaldehyde, permeabilized with 100% ice-cold methanol, and incubated with 10% normal goat serum containing 0.3% Triton™ X-100. After washing in *Tris*-based saline-Tween 20 (TBS-T), cells were incubated with a polyclonal anti-PTPRD antibody (LifeSpan BioSciences, Seattle, WA, USA) at a dilution of 1:100 at 4°C overnight, and subsequently with an FITC-conjugated secondary antibody (BioLegend, San Diego, CA, USA). Cell nuclei were counterstained with 4',6-diamidino-2-phenylindole (DAPI; Boster, Wuhan, China), and cells were then viewed under an Olympus BX41 fluorescence microscope and photographed using an Olympus DP72 camera (Olympus America, Central Valley, PA, USA).

### RNA isolation and quantitative RT-PCR (qRT-PCR)

Total cellular RNA was isolated using TRIzol Reagent (Invitrogen) and reversely transcribed into cDNA using TransScript^®^ One-Step gDNA Removal and cDNA Synthesis SuperMix (Tansgen Biotech, AT311, China) according to the manufacturers’ instructions. The resulting cDNA samples were subjected to qPCR amplification using the SYBR Premix Ex Taq II (TaKaRa, Dalian, China) assay kit in a 20 μl reaction mixture according to the manufacturer's instructions. The primers used were as follows: PTPRD, 5'-TCAGACAGACATTGCATCATCCAG-3' and 5'-GCCAAATGGGCAGTTATTGTTTC-3'; STAT3, 5'-CACATGCCACTTTGGTGTTTCA-3' and 5'-GGGCAATCTCCATTGGCTTC-3'; and GAPDH, 5′-GCACCGTCAAGGCTGAGAAC-3′ and 5′-TGGTGAAGACGCCAGTGGA-3′. After amplification, all samples were normalized to the internal control (GAPDH) and fold changes were calculated using relative quantification (RQ = 2^-ΔΔCT^). The experiments were done in triplicate and repeated at least once.

### Protein extraction and western blot

Total protein lysate from cells or tissues was prepared using standard RIPA lysis buffer (Sigma Chemicals, St. Louis, MO, USA). To minimize protein dephosphorylation, phosphatase-inhibitors (Santa Cruz Biotechnology, Santa Cruz, CA, USA) were added into the lysis buffer. Protein concentration was then measured using a bicinchoninic acid assay (Thermo Scientific, Bonn, Germany). Fifty to 80 μg of each protein lysate was separated by 8-12% sodium dodecyl sulfate-polyacrylamide gel electrophoresis (SDS-PAGE) and transferred onto polyvinylidene fluoride (PVDF) membranes (Millipore, Billerica, MA, USA). Membranes were then blocked in 5% skim milk solution in TBST for 1 h at room temperature and then incubated with primary antibodies raised against PTPRD (1:500; LifeSpan BioSciences), ALDH1 (1:300; Proteintech Group, Chicago, USA), STAT3 antibody (1:500; Proteintech Group), pSTAT3 (Tyr705) (1:500; Cell Signaling Technology, Inc., Danvers, MA, USA), OCT-4 (1:300; Proteintech Group), E-cadherin (1:500; Proteintech Group), and vimentin (1:500; Proteintech Group) at 4°C overnight, and subsequently with an IRDye 800 CW-labeled secondary antibody (1:5,000). Protein bands were quantified by optical density analysis and normalized to GAPDH.

### Wound-healing assay

After gene transfection, cells were grown to 70%-80% confluence in 6-cm cell culture dishes, and cell monolayers were scratched across the center using a 10 μl pipette tip. Cells were washed with DMEM twice and then cultured in DMEM without FBS for up to 72 h. Images were captured using an Olympus IX73 microscope connected to an Olympus DP73 camera at 0, 24, 48, and 72 h.

### Tumor cell migration and invasion assays

To analyze breast cancer cell invasion capacity, cell inserts (8.0 μm pore size membrane; Corning, Corning, NY, USA) were coated with 100 μl Matrigel (BD Bioscience, San Jose, CA, USA) diluted 1:3 in DMEM (for invasion assays), or left untreated (for migration assays). Subsequently, 2 × 10^4^ cells, previously cultured in low FBS (0.1%) medium for 24 h, were seeded onto cell inserts in the upper chambers in serum-free medium. The lower chamber was filled with 0.5 ml DMEM containing 10% FBS. After culturing for 24 h, migrating or invading cells in the lower surface of the membranes were fixed with methanol, stained with 0.01% crystal violet and photographed. Results represent the average number of cells in 5 fields per membrane in triplicate inserts.

### Isolation of CD44^+^/CD24^−^ breast cancer stem cells

MDA-MB231 cells were starved for 24 h in DMEM/F12 without FBS and dissociated into single-cell suspensions using 2.5% trypsin followed by centrifugation at 300 g for 5 min. Cell pellets were resuspended in 40 μl of PBE suspension buffer (for approximately 1× 10^7^ cells) and incubated with CD24 microbeads (CD24 Microbead Kit; Miltenyi Biotec, Bergisch Gladbach, Germany) followed by a magnetic separation. CD24^−^ cells were collected, washed, and incubated with CD44 microbeads (Miltenyi Biotec) at 4°C for 15 min. After washing and resuspension in 500 μl of PBE buffer, magnetic separation was used for enrichment of CD44^+^/CD24^−^ cells. To detect the effect of IL-6 on STAT3 signaling in BCSCs and non-BCSCs, before isolation MDA-MB-231 cells were cultured in DMEM for 24 h and then cultured again in DMEM/F12 containing 20 ng/ml of IL-6.

### Tumor cell colony formation assay

BCSCs were isolated using magnetic beads and seeded into 6-well plates in triplicate at a density of 500 cells/well. After culturing for 14 days in complete medium (changed every 3 days), the cells were fixed with methanol and stained with 0.1% crystal violet. Cultures were then photographed and cell colonies were counted and measured using Image-Pro Plus image analysis software (Media Cybernetics, Inc., Rockville, MD, USA).

### Mammosphere formation assay

After transfection with PTPRD siRNA or NC siRNA for 24 h, BCSCs were isolated, inoculated into ultra-low attachment 6-well plates (Corning) at a density of 4 × 10^4^ cells/well, and grown in DMEM/F12 supplemented with B27 (1:50, Invitrogen), 20 ng/ml human recombinant EGF (Sigma-Aldrich), 20 ng/ml bFGF (Sigma-Aldrich), 4 μg/ml heparin (Sigma-Aldrich), and 5 μg/ml insulin (Sigma-Aldrich) for 14 days. Cell colonies larger than 60 μm in diameter were counted under an inverted microscope (Olympus Corporation, Tokyo, Japan).

### Flow cytometry analysis of the CD44^+^/CD24^−^ cell population

After isolation, cells were diluted at a density of 10^6^ cells/ml and incubated with anti-human CD44-FITC and CD24-PE antibodies (BioLegend, San Diego, CA, USA) at 4°C for 30 min. Flow cytometric analysis was performed using a FACSCalibur Flow Cytometer (BD, Franklin Lakes, NJ, USA).

### Cell viability assay

Cell viability was measured using the Cell Counting Kit-8 (CCK-8) assay kit (Dojindo, Kumamoto, Japan). In brief, cells were transfected with PTPRD siRNA or NC siRNA for 24 h and then sub-cultured for up to 72 h. At the end of each experiment, 10 μl of CCK-8 solution was added into each well and the cells were further incubated at 37°C for 2 h. Absorbance was measured in a microplate reader at 450 nm. DMEM/F12 was used as a blank control. Proliferation data were expressed as % of NC control.

### Mouse xenograft model

Animal experiments and procedures were approved by the Animal Care and Use Committee of Dalian Medical University and performed in accordance with the Guide for the Care and Use of Laboratory Animals (NIH). Ten severe combined immunodeficiency (SCID) mice were randomly divided into two groups. Negative control shRNA (shRNA-NC) or PTPRD shRNA-transfected MDA-MB-231 cells (5×10^5^) were resuspended in 100 μl phosphate buffered saline (PBS) and injected into mammary fat pads. Tumor volumes (V) were monitored and recorded every other day. On day 28 after tumor cell inoculation, mice were sacrificed and tumors were excised and analyzed by immunohistochemistry and western blotting.

### Statistical analysis

All *in vitro* experiments were performed in triplicate and repeated at least three times. Data were expressed as mean value ± standard error (SEM). Statistical analysis was performed using SPSS version 19.0 (SPSS, Chicago, IL, USA) or GraphPad Prism 5 (GraphPad Software, La Jolla, CA, USA). All other data were analyzed using unpaired two-tailed Student's *t*-test. *P* < 0.05 was considered statistically significant.

### Ethical approval

This study was reviewed and approved by the Ethical Committee and Institutional Review Board of Dalian Medical University.

## SUPPLEMENTARY MATERIALS FIGURES AND TABLES



## Abbreviations

BCSC: breast cancer stem cells
CSC: cancer stem cells
PTPRD: Receptor-type tyrosine-protein phosphatase δ
PTP: protein tyrosine phosphatase
STAT3: Signal transducer and activator of transcription 3
IL-6: interleukin-6
CD44: cluster of differentiation 44, standard isoform
CD24: cluster of differentiation 24, standard isoform
CSC: cancer stem cell
EGFR: epidermal growth factor receptor
EMT: epithelial-mesenchymal transition
FGF: fibroblast growth factor
HGF/SF: hepatocyte growth factor/scatter factor
shRNA: small hairpin ribonucleic acid
siRNA: small interference ribonucleic acid
ALDH1: aldehyde dehydrogenase 1
OCT-4: Octamer-binding transcription factor-4
