# Supplementary material for: Protein tyrosine phosphatase receptor-type δ acts as a negative regulator suppressing breast cancer
Source: Oncotarget. 2017 Oct 24;8(58):98798–811. doi: 10.18632/oncotarget.22000 (PMC5716768; doi:10.18632/oncotarget.22000)
Supplement: Supplementary file 1 [file oncotarget-08-98798-s001.pdf]

Protein tyrosine phosphatase receptor-type δ acts as a negative regulator suppressing breast cancer

SUPPLEMENTARY MATERIALS

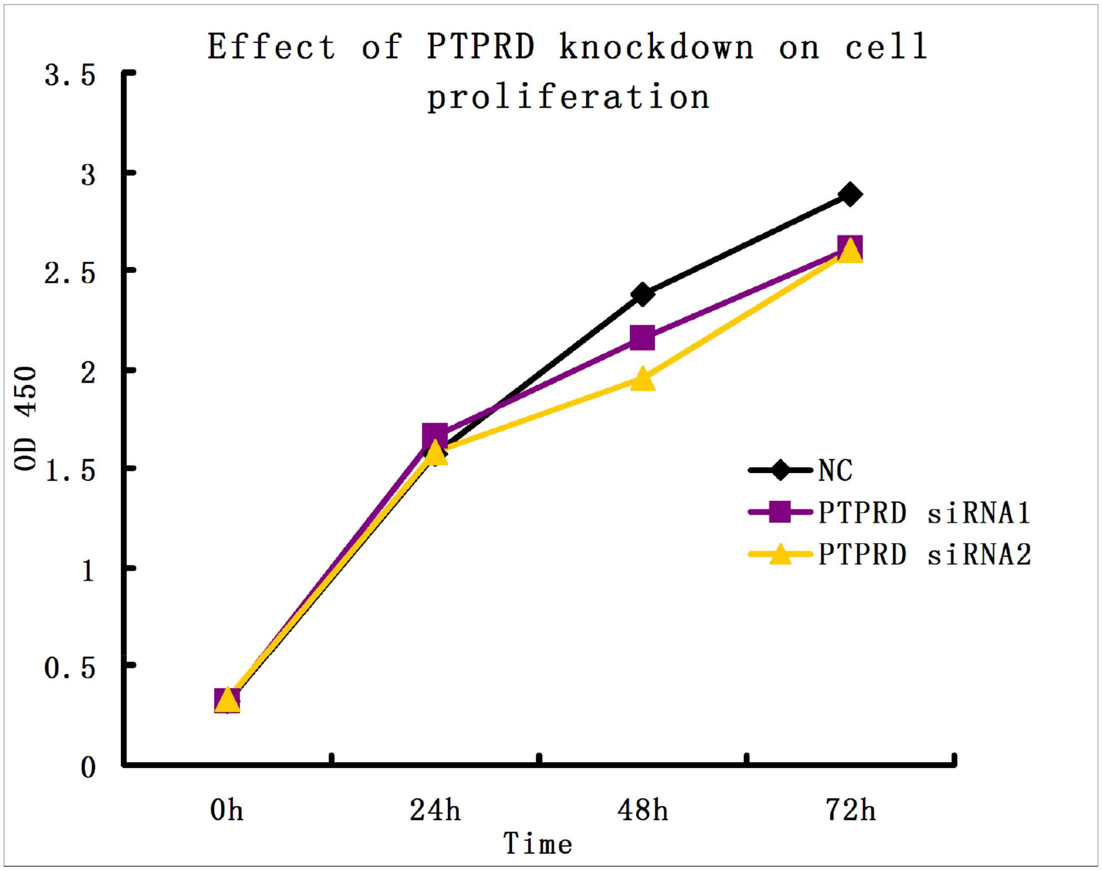

Supplementary Figure 1: Proliferation (CCK 8) assay in MDA-MB-231 cells transfected with NC or PTPRD siRNAs. No significant differences in cell proliferation rates were detected between the PTPRD siRNA and the NC siRNA groups.

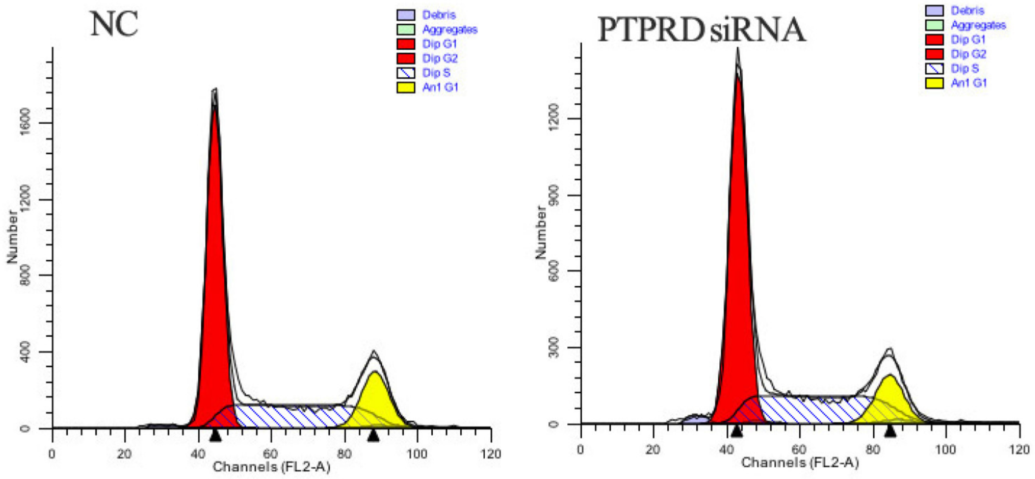

Supplementary Figure 2: Cell cycle staging evaluation in MDA-MB-231 cells transfected with NC or PTPRD siRNAs. PI (Propidium iodide)-stained cells were analyzed by fluorescence-activated cell sorting. No significant differences in cell cycle distribution between the two groups were found.
